# DAIR to Be Different: Successful Use of DAIR Regimen, a Novel Treatment Combination for EBV-induced HLH

**DOI:** 10.46989/001c.143659

**Published:** 2025-10-08

**Authors:** Adam Bouhadana, Amir Steinberg

**Affiliations:** 1 New York Medical College, 40 Sunshine Cottage Rd, Valhalla, NY 10595, United States; 2 Hematology and Oncology, Westchester Medical Center, 100 Woods Rd, Valhalla, NY 10595, United States

**Keywords:** HLH, sHLH, case, EBV-induced HLH, Hyperinflammatory syndrome

## Abstract

Hemophagocytic lymphohistiocytosis (HLH) is a life-threatening hyperinflammatory syndrome caused by uncontrolled immune activation and hemophagocytosis. In adults, Epstein-Barr virus (EBV) infection is a known trigger. This report describes a novel approach to treating EBV-induced HLH in a 29-year-old female, avoiding the potential toxicities of chemotherapy-based regimens, such as infertility. The patient presented febrile, tachycardic, and hypotensive following a week of high fever, bilious emesis, and diarrhea. Laboratory results showed elevated AST and ALT, hyperbilirubinemia, leukopenia, thrombocytopenia, and hyperferritinemia. Imaging revealed splenomegaly, and a positive mononucleosis rapid test confirmed EBV infection. Based on her clinical presentation, laboratory findings, and a bone marrow biopsy showing phagocytic histiocytes, she was diagnosed with EBV-induced HLH, fulfilling 7 of the 8 diagnostic criteria (5 required).

The patient was started on the DAIR regimen two days after transfer. DAIR combines dexamethasone for its potent anti-inflammatory properties (used in HLH-94/HLH-2004), anakinra to control the cytokine storm, IVIG for passive immune support and antiviral action, and rituximab to target EBV-infected B cells. The patient responded well to treatment. EBV became undetectable within three weeks, and by seven weeks, blood counts, liver function, ferritin, triglycerides, and fibrinogen levels had normalized. Ten weeks after symptom onset, she returned to work and is now pregnant, with no signs of relapse.

This case demonstrates the effectiveness of DAIR as a chemotherapy-free alternative for EBV-induced HLH, offering a targeted approach to the syndrome’s complex pathogenesis while achieving sustained remission.

Epstein-Barr virus (EBV) infection is a significant cause of hemophagocytic lymphohistiocytosis (HLH), a life-threatening hyperinflammatory syndrome characterized by uncontrolled immune activation and hemophagocytic activity by macrophages.^1^ This syndrome can be primary (pHLH), arising from genetic mutations, or secondary (sHLH) triggered by bacterial or viral infections like EBV, malignancy, or autoimmune disorder.^2^ In adults, EBV-induced HLH is a particularly challenging clinical entity due to its delayed diagnosis and fulminant course.^3^

The current treatment protocol includes the HLH-94 and HLH-2004 trials, consisting of etoposide, dexamethasone, and cyclosporine A. These advancements from HLH-94 and HLH-2004 have laid the groundwork for ongoing research into novel treatment strategies for pHLH.^4^

However, regarding sHLH, the HLH-94 and HLH-2004 guidelines have shown minimal efficacy in treatment outcomes and long-term survival.^5,6^ There are no definitive guidelines for sHLH, so its treatment usually targets the underlying trigger, considering the heterogeneity of its etiologies.

Additionally, traditional chemotherapy regimens can have significant side effects. They may not be optimal for all patients, and recent research has explored alternative therapeutic approaches targeting specific aspects of the HLH inflammatory cascade, offering the potential for improved treatment outcomes with reduced toxicity.

This paper presents a unique case of EBV-induced HLH successfully treated with a novel DAIR regimen. This regimen departs from conventional chemotherapy and uses a combination of targeted therapies:

- Dexamethasone for its potent anti-inflammatory effects, as used in HLH-94 and HLH-2004
- Anakinra, an interleukin-1 receptor antagonist, specifically targets a key inflammatory pathway in HLH pathogenesis to dampen cytokine storms.
- Intravenous immunoglobulin (IVIG) provides passive immune support, potentially modulates the immune response, and helps against the EBV virus.
- Rituximab, a B-cell-depleting monoclonal antibody, is aimed at directly eliminating EBV-infected B cells, the underlying source of the hyperinflammatory state.

There are documented cases demonstrating the effectiveness of each individual agent. That case describes a 22 year old female with EBV-induced HLH successfully treated with a combination of methylprednisone, anakinra, IVIG, and rituximab, which led to rapid clinical and virologic recovery.^7^ One study describes a combination of IVIG, anakinra and corticosteroids resulting in a 50% survival in eight critically ill patients with HLH. The patients in the study had various degrees of organ dysfunction and the need for aggressive ICU treatment.^8^

By discussing this case and the rationale behind the DAIR regimen, this paper aims to contribute to the evolving understanding of treatment options for EBV-induced HLH. We explore the potential benefits of this targeted approach in effectively controlling the hyperinflammatory state and eliminating the viral trigger, potentially leading to improved outcomes for patients with this complex and life-threatening condition.

The patient, a 29-year-old female, presented to the ED febrile, tachycardic, and hypotensive after a prolonged high fever, bilious emesis, and watery diarrhea ongoing for 1 week before arrival. The patient stated goals for pregnancy in the future. Laboratory investigations revealed elevated AST and ALT, hyperbilirubinemia of 1.7 g/dL, leukopenia of 1.53 x109/L, thrombocytopenia of 32 x 109/L, and ferritin of 10,800 ug/L. The sonogram revealed an enlarged spleen. A positive infectious mononucleosis rapid test confirmed EBV infection. Based on clinical presentation, laboratory findings, and EBV positivity, the patient was transferred to our institution for diagnosis of EBV-induced HLH. Bone marrow biopsy noted phagocytic histiocytes. IL2 Receptor (CD25) was 11,868 pg/mL. triglycerides 275 mg/dL, fibrinogen 113 mg/dL, EBV PCR was 28,800 international units/mL. She fulfilled 7 of the 8 criteria (5 needed) for diagnosing HLH, using the HLH-2004 criteria **(Table 1)** and she had a calculated H-score of 284, which correlates to >99% probability of hemophagocytic syndrome, using the H-score criteria.^9,10^ NK cell activity was requested though results were not obtained.

**Table 1.**
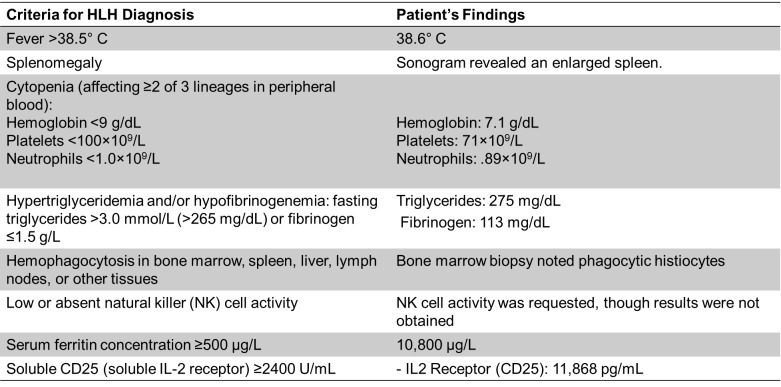
Patient’s Findings Compared to HLH 2004 Diagnosis Criteria

Intervention: The DAIR regimen was administered 2 days after the hospital transfer. DAIR consisted of:

- Dexamethasone (20 mg/day for 8 days starting on day 2)
- Anakinra (100 mg for 7 days, starting on day 3)
- IVIG (20g over five days, starting on day 2)
- Rituximab (375 mg/m2 for 4 weeks starting on day 2)

The patient responded remarkably well to the DAIR regimen. She reported improvement of symptoms almost immediately after starting treatment. Hematological recovery was also swift. By 7 weeks, the patient’s complete blood count (CBC) showed normalization or near normalization of previously low cell counts. After 10 days of treatment, liver function tests returned to normal ranges. Triglycerides, ferritin, and fibrinogen levels were also normalized. The patient tolerated treatment well, with no remarkable side effects observed throughout its course. Importantly, the patient has not exhibited any signs or symptoms suggestive of relapse since treatment completion, and she successfully returned to work 10 weeks after presentation. At the patient’s six month follow up, she reported feeling great and back to her baseline. She presented to this follow-up pregnant for 2.5 months.

Triglycerides were markedly elevated at baseline at 275 mg/dL peaked shortly after DAIR initiation, followed by a rapid and sustained decline to well below the generalized normal high by day 19, remaining stable thereafter. The patient experienced a 75.6% decrease in triglyceride levels from onset of the DAIR regimen to her six-month follow-up. **(Figure 1 (A))**

**Figure 1.**
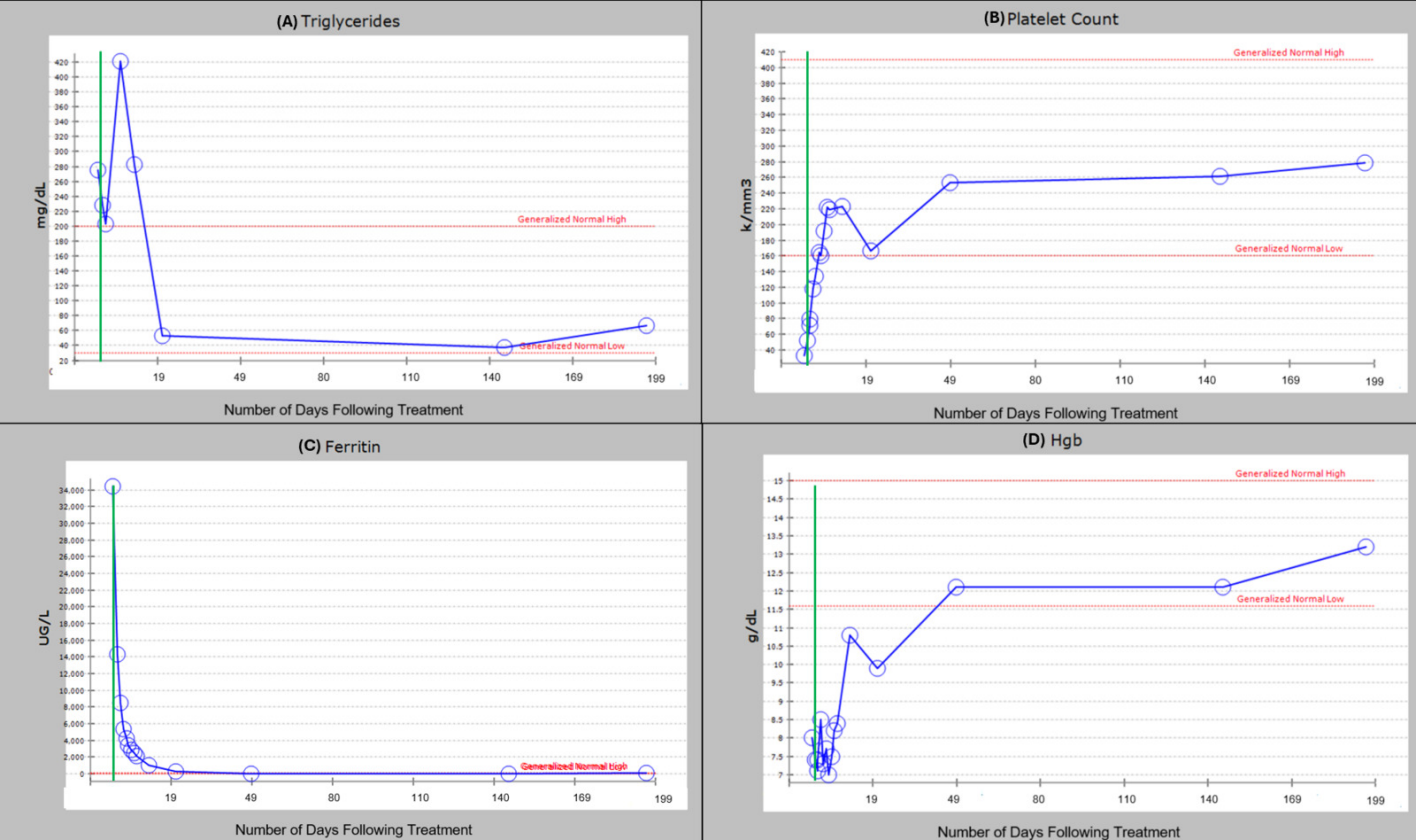
(A–D) Laboratory marker trends from Day 0 to Day 199, following initiation of the DAIR regimen (green vertical line). (A) Triglycerides (mg/dL), (B) Platelet Count (K/µL), (C) Ferritin (µg/L), and (D) Hemoglobin (g/dL) are plotted over time since treatment started. Red dashed lines represent generalized normal high and low reference ranges.

Platelets were initially low but progressively increased after treatment, entering the normal range by day 49 and continuing to rise to day 199. This represented a 687.9% increase in platelet count from onset of the DAIR regimen to her six-month follow-up. **(Figure 1 (B))**

Extremely elevated ferritin levels dropped precipitously following DAIR initiation, approaching normal levels by day 19, and decreasing 99.8% to her six-month follow-up. **(Figure 1 (C))**

Hemoglobin was initially low but showed a steady upward trend post-treatment, crossing the generalized lower normal limit by day 49 and continuing to increase 65% through day 199. **(Figure 1 (D))**

This case report presents a patient with EBV-induced HLH successfully treated with the DAIR regimen. The rapid decline in EBV viral load and normalization of laboratory findings suggest the potential efficacy of this approach.

The DAIR regimen offers several theoretical advantages over traditional chemotherapy regimens used for HLH. As a young woman of childbearing age, the risks associated with chemotherapy, including ovarian follicle depletion and infertility, were significant considerations.^11^ The current FDA label for etoposide phosphate states that it may cause infertility and result in amenorrhea and premature menopause.^12^ The patient in this case has become pregnant, alluding to the successful avoidance of potential infertility due to chemotherapeutic agents. Additionally, the use of etoposide in treating sHLH has been questioned due to its efficacy and many side effects, including myelosuppression, hepatotoxicity, and teratogenic potential.^13^ These risks necessitated an alternative approach that maintained efficacy while mitigating toxicity. No agent in the DAIR regimen is an established cause of female infertility.^14–16^

Dexamethasone, a glucocorticoid, was selected for its potent anti-inflammatory and immunosuppressive properties, which are critical in curbing the hyperinflammatory state characteristic of HLH. Dexamethasone’s role as a cornerstone in the HLH-94 and HLH-2004 guidelines further establishes its efficacy and reliability.^1,4^

Anakinra, an interleukin-1 (IL-1) receptor antagonist generally used in adult patients with rheumatoid arthritis, targets a key inflammatory pathway in HLH pathogenesis. Elevated IL-1 levels are a hallmark of hyperinflammatory conditions, and by blocking the IL-1 receptor, anakinra interrupts this critical inflammatory pathway commonly seen in sHLH.^17^ One retrospective study showed 90.5% fever resolution among patients with sHLH treated with anakinra.^18^ Another retrospective analysis showed that it is well tolerated and produces good responses to sHLH, with a higher response rate and longer survival than HLH-94 when paired with high-dose steroids, such as dexamethasone.^19^

IVIG was administered to provide passive immunity. A key advantage of IVIG is its ability to act systemically without directly inducing cellular cytotoxicity, making it particularly useful in pediatric and fertility-sensitive populations. A retrospective cohort study showed comparable efficacy between IVIG/dexamethasone combinations and the HLH-2004 protocol further supporting its inclusion, suggesting that etoposide and its induced toxicity in the HLH-2004 regimen can be avoided in sHLH patients.^20^

Rituximab, a CD20-targeting monoclonal antibody, was used to address the EBV-driven component of this patient’s HLH directly. By depleting EBV-infected B cells, rituximab reduces the viral load and mitigates the immune system’s hyperactivation in response to EBV antigens. This targeted mechanism addresses the patient’s disease’s primary driver and avoids traditional chemotherapy’s broad, nonspecific cytotoxic effects.^21^

Potential side effects of this combination include increased susceptibility to infections, cytopenia, and infusion-related reactions. Dexamethasone may cause hyperglycemia, hypertension, and mood disturbances; anakinra is associated with injection site reactions and neutropenia; IVIG can cause headache, fever, and, rarely, thromboembolic events or hemolysis; and rituximab carries risks of infusion reactions, hypogammaglobulinemia, and viral reactivation.^22–25^ However, the patient tolerated therapy well, with no remarkable side effects observed during or after treatment.

Compared to conventional chemotherapy, the DAIR regimen offers a more tailored approach with potentially fewer side effects. However, further research is needed to evaluate its long-term safety and efficacy in a larger patient population. This case highlights the potential of the DAIR regimen as a promising alternative for managing EBV-induced HLH, particularly in patients for whom traditional chemotherapy poses significant risks.

This is a single case report, and the findings cannot be generalized to the broader population with EBV-induced HLH. Further studies with larger patient cohorts are necessary to confirm the efficacy and safety of the DAIR regimen.

## Authors’ Contribution

Conceptualization: Amir Steinberg

Data curation: Amir Steinberg, Adam Bouhadana

Formal Analysis: Amir Steinberg, Adam Bouhadana

Supervision: Amir Steinberg

Writing – original draft: Adam Bouhadana

Writing – review & editing: Adam Bouhadana, Amir Steinberg

## Competing Interest – COPE

No competing interests were disclosed.

## Ethical Conduct Approval – Helsinki – IACUC

The patient provided informed consent. This study follows institutional guidelines. Per the institution, a single case report does not require an IRB submission.

## Informed Consent Statement

All authors and institutions have confirmed this manuscript for publication.

## Data Availability Statement

All are available upon reasonable request.
